# The Role of Al-10%Si Coating in the Manufacture and Use of Aluminized Open-Joint Steel Tubes

**DOI:** 10.3390/ma15124210

**Published:** 2022-06-14

**Authors:** Krzysztof Żaba, Tomasz Trzepieciński

**Affiliations:** 1 Department of Metal Working, Physical Metallurgy of Non-Ferrous Metals, Faculty of Non-Ferrous Metals, AGH—University of Science and Technology, 30-059 Cracow, Poland; 2 Department of Manufacturing and Production Engineering, Faculty of Mechanical Engineering and Aeronautics, Rzeszow University of Technology, 35-959 Rzeszów, Poland; tomtrz@prz.edu.pl

**Keywords:** Al-Si coating, open-joint aluminized steel tubes, annealing, SEM

## Abstract

The paper presents the results of laboratory and industrial tests on the role of Al-10%Si coatings in the manufacture and use of welded aluminized steel tubes. The tubes were fabricated from DX53D + AS120 steel tubes coated with Al-10%Si coating. Investigations were carried out on individual processes in the manufacture of welded tubes aimed at determining the effect of the coating properties on the conditions of the forming process and vice versa. In the next step, a quality assessment has been conducted on the finished tubes. Then, selected tests simulating the operating conditions of tubes used for the elements of exhaust systems are presented. Analyses of the susceptibility of strips to plastic deformation and evaluation of the adhesion of the Al-10%Si coating to the steel base metal were carried out using bending tests. From the results, it was proved that the Al-10%Si coating determines the technical features of the workpiece material, affects the manufacturing process, and determines the quality of aluminized steel tubes. The quality of the coating surface depended on the annealing temperature and annealing time. The higher the temperature, the shorter the time needed to produce a change in the coating properties. A *p*-value reported from a tests is less than 0.05.

## 1. Introduction

One of the products manufactured for the automotive industry is a steel tube used for elements of the exhaust systems of motor vehicles. Hot rolled steel strip wound in coils is one of the feedstocks used in the production of tubes. This is of a width and thickness dependent on the diameter and thickness of the finished tube wall, coated on both sides with Al-Si. Information on the problems of production and the properties of the coating has been presented in [1,2,3,4,5,6,7,8,9,10,11,12,13] among other sources.

The issues related to the production and properties of the Al-Si coating on a steel substrate have formed a research area that has proved very popular among researchers for many years. In particular, this includes research on the structure of the Al-Si coating after the process of hot-dipped aluminizing [14,15], including the influence of Si [16,17,18,19,20,21], but also Cu [22,23,24], Cr [25], Re [26,27], La [28,29], Ce [30], Mo, W and Nb [31]. The addition of Cu increases the corrosion resistance of the Al-Si coating. In the range of 2.5–11%, Cu causes a reduction in phase growth. However, above 15% Cu does not reduce the thickness of the alloy layer, but it transforms the boundary between the intermetallic layer and the substrate from irregular to smooth. The addition of 1% Cr to molten aluminum causes the formation of the Al_13_Cr_2_ phase in the intermetallic coating, replacing the Al_5_Fe_4_ phase. The addition of 0.1–0.5% rare earth metals makes the structure of the coating more homogeneous and the dividing plane between the substrate and the alloy layer smoother. The addition of Mo, W or Nb reduces the thickness of the intermetallic layer. The primary purpose of adding Ti is to accelerate the alloying of the coating and to prevent the formation of voids, thereby preventing flaking and improving the oxidation resistance of the alloyed coatings. A number of works concern the structure of the coating after heat treatment [32,33,34,35,36,37,38,39,40,41], corrosion resistance [42,43,44,45,46,47], and susceptibility to plastic shaping [48,49,50].

The reason for the use of such material is a combination of the strength and plastic properties of steel with the high corrosion resistance [51] of the Al-Si coating coupled with commercially reasonable costs of production and maintenance of the tubes. As is well known, tubes are produced in a continuous forming process from a strip, with welding and calibration due to the uniformity desired in the final product. This is obtained through the process of bending and expanding tubes which must meet strict requirements for tolerances in diameter, wall thickness and mechanical properties [52,53].

The presence of Al-Si coating is very important since it affects the quality of the processes and consequently determines the quality of the tubes. An Al-Si coating may introduce risks associated with the presence of the components in the welding zone, which may lead to impurity of the connector between aluminum and silicon oxides at high temperatures, and consequently the appearance of defects during the manufacturing or processing of tubes and failure during the operation of the vehicle.

There is general information in the literature specifying ranges of parameters in the production of welded steel tubes [54,55,56,57,58,59] and Friction Stir Welding [60,61]. By contrast, knowledge on the preparation of aluminized steel tube is incomplete, and in particular in the area related to the presence of the coating. Although scientists are researching aluminized steel strips, the area related to aluminized steel pipes is virtually omitted in the publications. The analysis of the influence of parameters on the pipe manufacturing process, including the Al-Si coating, is not available in the world literature. Therefore, the research presented in the publication is a kind of novelty in this area. It was therefore decided to carry out experimental studies to examine the influence of the Al-10%Si coating on the manufacturing process and the quality of the aluminized steel tubes. As part of the so-defined objective, tests were performed in the following thematic groups:

1. Research on the properties and microstructure of the Al-10%Si coating applied to the steel strip.
2. Research on the individual processes for producing welded steel tubes coated with Al-10%Si, with particular emphasis on the effect of the coating on the processes and the processes on the coating.
3. Research on the properties and microstructure of the Al-10%Si coating applied on the tubes and their effect on their susceptibility to plastic forming.
4. Model tests of pipes simulating operating conditions.

Reaching such a target objective was achieved through laboratory and industrial research with the use of advanced techniques for microscopic observation, microanalysis of the chemical composition, techniques for measuring the geometrical surface condition, mechanical properties and the use of statistical methods.

## 2. Materials and Methods

Steel strips in grade DX53D + AS120 with a thickness of 1.5 mm were chosen as test material. Both sides were coated with Al-10%Si with a thickness of about 18 µm on one side of the strip and a thickness of about 23 µm on the other side of the strip. The Al-10%Si coating was produced by continuous immersion in a bath with an approximate chemical composition of 10 wt.% Si and 90 wt.% Al. The requirements concerning continuous hot-dip coated DX53D steel flat products for cold forming defined in standard [62] are shown in Table 1 and Table 2.

As part of the study, the stabilities of the chemical composition and thickness of the strips were determined. Research was performed on the mechanical properties of the strip, its susceptibility to plastic forming and adhesion of the coating to the steel substrate. Microscopic observations of the coating surface were obtained. These observations were made for the cross-section of the coating as well as point and linear microanalysis of its chemical composition. Surface roughness, both thicknesses and the microhardness of the coating were determined.

**Figure 1:**
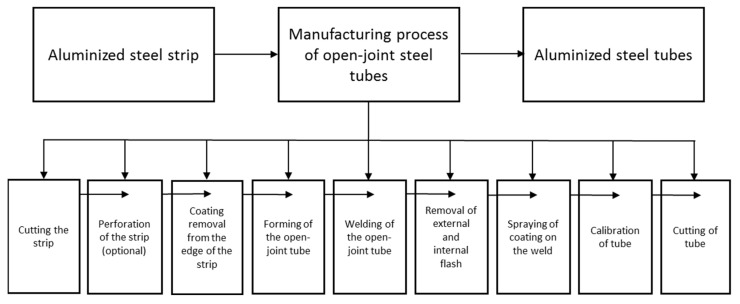
Diagram of the manufacturing process of aluminized steel pipes.

In the next stage, investigations were carried out on the individual production processes of the welded tubes made of galvanized steel strips. The study was targeted at determining the influence of the Al-10%Si coating on the production processes and the influence of the process parameters on the properties of the Al-10%Si coating, which translates into tube quality. A diagram of the production of welded steel pipes with the Al-10%Si coating is shown in Figure 1.

The manufacture of open-joint tubes begins with cutting the tape into strips of appropriate width, resulting from the diameter of the tube, the technological margin of the material on the flash during welding and the need for final calibration of the tube. Open-joint tubes are formed in a continuous system driven by forming rollers with horizontal axes and not driven rollers with vertical axes. Welding takes place in the system of pressure rollers. The edges of the open-joint tubes are heated by high-frequency currents, induced by the inductor which surrounds the tube. The internal flash is removed by a special knife.

The next unitary process for the production of welded pipes is replenishment of the coating in the weld area after removing the external flash. This process is commonly used due to the basic corrosion resistance requirements for pipes intended for exhaust system components. The coating is replenished by a thermo-mechanical method, using the dynamics of the flue gas stream in an acetylene burner using Sulzer Metco thermal spray coating equipment. To replenish the coating, a metallizing wire with a diameter of 2.7 mm with the chemical composition shown in Table 3 is used. The wire is fed to the spray gun in a continuous fashion.

The wire consumption was 80 mm/min, with a feed speed of 60 m/min. After the wire was melted in the spray gun, the material was sprayed onto the surface of the tube in the area of the weld where the outer flash has been removed. The process was carried out continuously.

The next step is calibrating, whose role is primarily to give the tubes a circular shape. The final step is to cut the tubes to an appropriate size and pack them. All relevant processing parameters of the material are controlled. Cutting edge quality was tested in the process of cutting coils. Microscopic observations and microanalysis of the chemical composition of the cutting edge were made in order to identify the coating quality during cutting.

In the process of removing the coating from the edge of the strip, microscopic observations, microanalysis of the chemical composition at strip edges after the process and energy-dispersive X-ray spectroscopy (EDS) mapping was carried out. The aim of the study was to evaluate the effectiveness of this process by identifying any residual coating on the edges of the strip. In the open-joint tube forming process, investigations of the thickness and surface roughness of the coating were performed. The measurements were carried out in a perpendicular direction to the tube axis in the areas in which the strip was in contact with the rollers. In the welding process microscopic observation of the join zone, micro-hardness measurements, linear microanalysis of the chemical composition and EDS mapping in several cross-sections of the weld were carried out in order to detect the presence of aluminum and silicon.

The evaluation of the quality of the removal process of the external and internal flash was made by macroscopic and microscopic observations. In the process of filling, the coating macroscopic and microscopic examination and EDS mapping of the coating deposited were performed. Adhesion tests of the coating deposited on conically expanded tubes were carried out. The coating thickness and its surface roughness were also examined in the calibration process. Surface roughness measurements were made in a direction perpendicular to the tube axis in the areas in which the formed strip is in contact with the rollers. Additionally, an evaluation of the calibration process was carried out on the basis of measurements of the diameter and thickness of the tube wall.

Finished tubes with 50 mm outer diameter and a wall thickness of 1.5 mm were evaluated for quality and susceptibility to plastic forming. The requirements concerning for welded tubes are shown in Table 4.

Measurements were made of the diameter, wall thickness of the tube and height of the flash. Research was carried out on the mechanical properties and expansion on the cone tube section. After each examination of the susceptibility of the tube to plastic forming, the adhesion of the Al-10%Si coating to the base was evaluated. In the next step, macroscopic and microscopic observations of the coating surface of the tubes were performed as well as evaluations of their thickness, surface roughness and micro-hardness.

In model tests simulating the operating conditions of aluminized steel tubes used for elements of exhaust systems, macroscopic and microscopic observations of the condition of the coating surface, surface roughness, wall thickness, and chemical composition were made, and measurements of the coating structure were performed after annealing at a temperatures of 250–700 °C for 30, 180 and 1440 min. The surfaces of the Al-Si coating on the strip and pipes were subjected to macroscopic observations. For this purpose, a Nikon D80 digital camera was used. The surfaces of the Al-10%Si coating on the strip and tubes were subjected to macroscopic observation using a Nikon Multizoom AZ 100 optical microscope.

Microstructure observations were carried out on metallographic sections made from samples using the Struers Rotopol 11 device (Struers, Copenhaga, Denmark). The samples were quenched in Epofix Struers resin, and then polished using abrasive papers with a gradation 220–1000 µm and polished on polishing shields with diamond suspension with a gradation 15, 9, 6, 3 and 1 µm. Observations of the Al-10%Si coating microstructure, the intermetallic layer (IL) of the Al-Fe-Si and the base metal were performed using a Hitachi 3500 N scanning electron microscope (Hitachi Ltd., Tokyo, Japan). The diameter of the electron beam was about 0.01 µm.

In order to determine the chemical composition of the Al-10%Si coating and base metal, point and linear microanalyses were carried out on samples intended for microstructural investigations using a Hitachi FE-SEM SU-70 scanning electron microscope (Hitachi Ltd., Tokyo, Japan) equipped with a Thermo Scientific Noran System for analysis of chemical composition by X-ray dispersion. Selected areas of the coating were also mapped with the SU-70 microscope.

**Figure 2:**
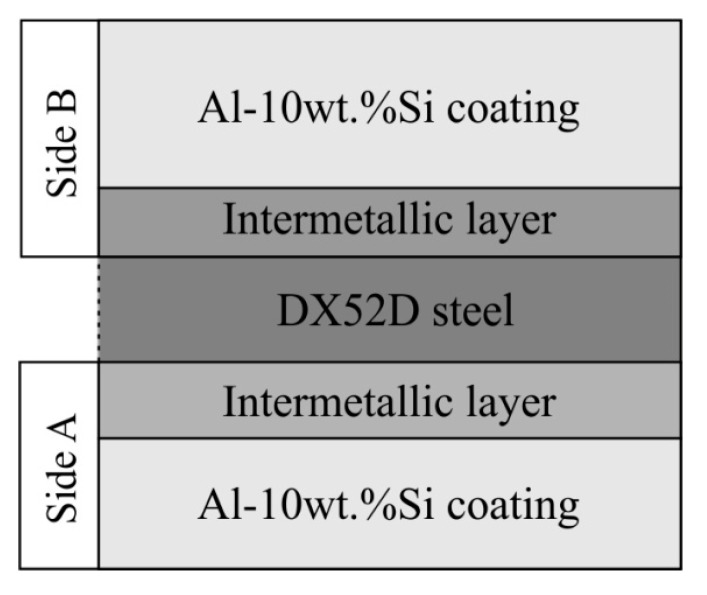
Schematic cross-section of the aluminised steel strip with the sides of measurement of the thickness of the Al-10%Si coating assigned.

Non-destructive electromagnetic measurements of the Al-10%Si coating thickness were performed using a calibrated Elkometer 345 instrument t (Elcometer Limited, Manchester, UK). Coating thickness was measured on both sides of the strips. Sides of the sample were marked as A for the thinner coating thickness and as B for the thicker Al-10%Si coating thickness (Figure 2). During the manufacturing process of the tube and on the products, coating thickness was only measured on the outer surface of the coating (side B).

Measurements of surface roughness of the Al-10%Si coating were performed by the contact method using a Surfom 130A device (Zeiss Industrial Metrology, Warsaw, Poland) equipped with a Zeiss photo-optical head. Thirty measurements were made on each side of the strip sample (A and B) and average values of the roughness parameters of the coating surface, Ra and Rz, were obtained. Measurements were carried out according to the ASME B46.1:2009 standard [63].

Microhardness tests of the Al-10%Si coating and the base metal were carried out according to the ISO 4516:2002 standard [64] using a Shimadzu HMV-2 hardness tester (Shimadzu, Kyuto, Japan) equipped with a MicroVickers penetrator with 30 measurements being performed in total. The time of each measurement was 10 s. The load was F = 245.2 mN (HV0.025) for Al-10%Si coating tests and F = 19.614 N (HV2) for the base material.

Measurements of the strip and of the tube wall thickness were carried out using a Mitutoyo type E micrometer (Mitutoyo Corporation, Sakado, Japan) and a MiniTest ultrasonic wall thickness gauge (Electromatic Equipment Co., Lynbrook, NY, USA). Measurements of the diameters of the tubes were made using a Mitutoyo 30 digital caliper.

The mechanical properties of the strip were determined using a uniaxial tensile test according to the ISO 6892–1:2009 standard [65] on samples cut at angles of 0°, 45° and 90° in relation to the rolling direction. Stretching speed was set at 10 mm/min. Three samples were tested in each direction. As a result, the average values of the following parameters were determined: ultimate tensile strength (UTS), yield strength (YS_0.2_), uniform elongation A_r_ and total elongation A_80_.

**Figure 3:**
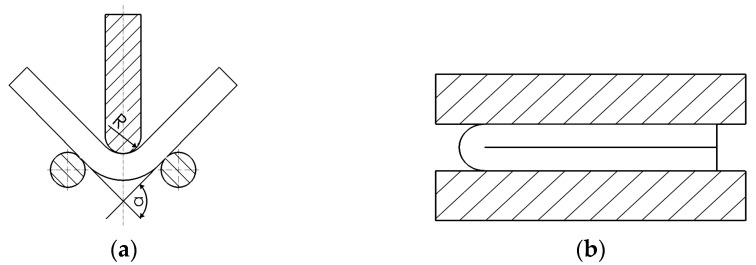
A method of testing susceptibility to deformation and coating adhesion at the bending angles (**a**) α = 90° and (**b**) α = 180°.

Analysis of the susceptibility of the strip to plastic deformation and evaluation of the adhesion of the Al-10%Si coating to the steel base metal were carried out using a bending test which can achieve a bending angle of 90° (Figure 3a).

Samples were subjected to the bending process using a universal testing machine. The speed of a punch with fillet radius R = 1.5 mm was 10 mm/min. Each test was carried out to bend the sample under an angle of 90°, as evidenced by increasing bending strength. Each test was repeated three times. Then, the specimens were bent to an angle of 180° (Figure 3b) between two parallel plates mounted on a universal testing machine. Bending speed was set at 10 mm/min. The adhesion of the coating was evaluated, in both cases, on the outer surface in the area of the bending radius.

**Figure 4:**
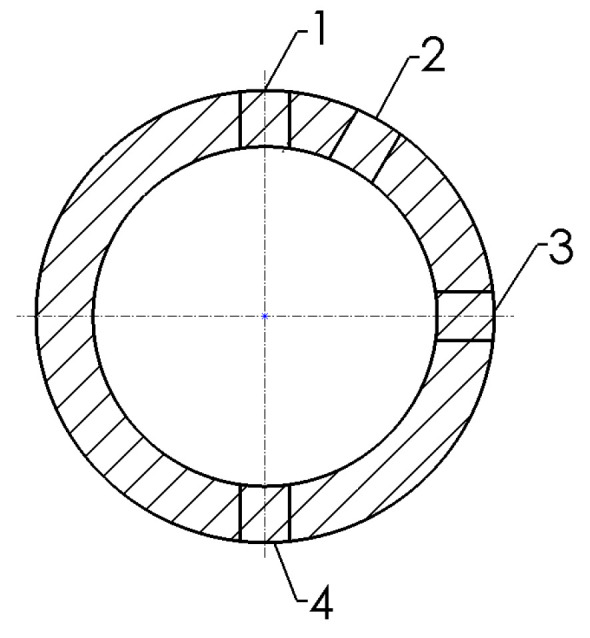
Areas of the tube, from which the test specimens for mechanical properties were cut; 1—welding zone, 2—30° from welding zone, 3—90° from welding zone, 4—180° from welding zone.

The mechanical properties of the tubes (Figure 4) were determined on samples in the form of strips with dimensions of 100 × 10 mm, cut from the weld (1) and from tube sectors located at an angle of 30° (2), 90° (3) and 180° (4) from the location of the weld. Tests on samples in the form of tube sections were also carried out. A uniaxial tensile test has been conducted according to [65]. Stretching speed was 10 mm/min. As a result of tests, the average values of the tensile strength UTS, yield strength YS_0.2_, uniform elongation A_r_ and total elongation A_80_ were obtained.

**Figure 5:**
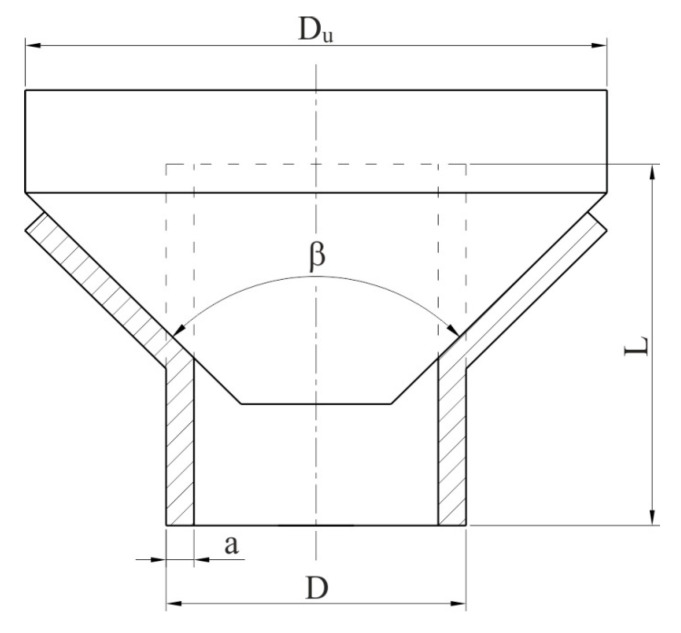
Schematic diagram of the expanding test [66].

The study of the height of the outer flash was performed using a Mitutoyo universal micrometer. In order to determine the tube susceptibility to plastic forming and coating adhesion, an expansion on the cone test (Figure 5) was performed according to [66]. For this purpose, three tube sections with a length of 30 mm were prepared, which were expanded using a cone-shaped tool made of 145Cr6 steel and with an angle of 90°. Coating adhesion on the tube’s outer surface was determined in the expanded area.

## 3. Results and Discussion

### 3.1. Properties of Aluminized Steel Strip

Because the results of the chemical composition of the substrate (Table 5) did not show deviation from the tolerance range of the content of individual elements, it can be concluded that the material meets the requirements in the context of its intended use on the welded tubes. The results of strip thickness measurements of the DX53D + AS120 are in the range g_t_ = 1.496–1.504 mm. The average value and the median values are approximately g_t_ = 1.5 mm. Considering the dimensional tolerances ±0.05 mm, the strip satisfies the thickness requirements.

The mechanical properties of the strip specimens slightly depend on the direction the specimen is cut in relation to the sheet rolling direction. Tensile strength in the sheet rolling direction reaches UTS_0_ = 290 MPa and increases for samples cut at an angle of 45° to the value UTS_45_ = 293 MPa and for samples cut at 90° reaches the lowest value UTS_90_ = 288 MPa. Similarly, the yield strength reaches its highest value for the samples cut at an angle of 45° YS_0.2-45_ = 172 MPa. An intermediate value was reached for samples collected at 90° YS_0.2-90_ = 171 MPa, and the smallest value was obtained for samples cut along the sheet rolling direction YS_0.2-0_ = 167 MPa.

The plastic properties evaluated by total elongation A_80_ and uniform elongation E_u_ do not depend on the sampling direction. The total elongation of the samples taken along the sheet rolling direction is A_80-0_ = 36%. This value decreases for samples cut at an angle of 45° to the value of A_80-45_ = 35%, and, for samples cut at an angle of 90°, it reaches the highest value of A_80-90_ = 37%. Similarly, the uniform elongation for directions of 0°, 45° and 90° reaches 23%, 21% and 23%, respectively. Regardless of the sampling direction, the mechanical properties of the aluminised steel strips are within the range specified in the requirements contained in Table 1.

**Figure 6:**
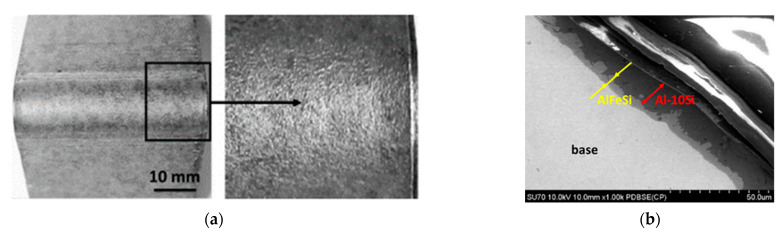
Macroscopic (**a**) and microscopic (**b**) observations of the coating surface after bending at an angle of 90° and SEM micrograph of base–coating interface.

**Figure 7:**
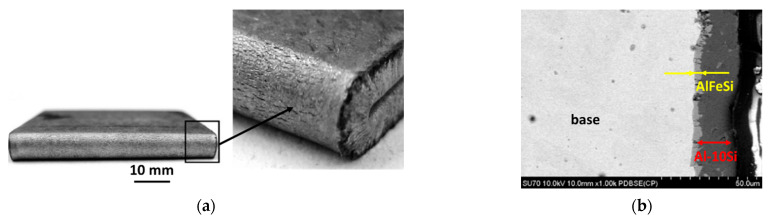
Macroscopic (**a**) and microscopic (**b**) observations of the coating surface after bending at an angle of 180° and SEM micrograph of base–coating interface.

Samples subjected to bending at 90° indicate no cracking in the cross-section of the material tested. On the outer surface, no changes in the form of coating exfoliation or tearing were observed (Figure 6). The results demonstrate that the samples are highly suitable for plastic forming and that there is good adhesion of the coating to the substrate. A similar situation applies to samples subjected to bending at an angle of 180° because no cracks in the cross section of the material were revealed. In contrast, small cracks occurred on the outer surface of the coating parallel to the bending axis (Figure 7).

**Figure 8:**
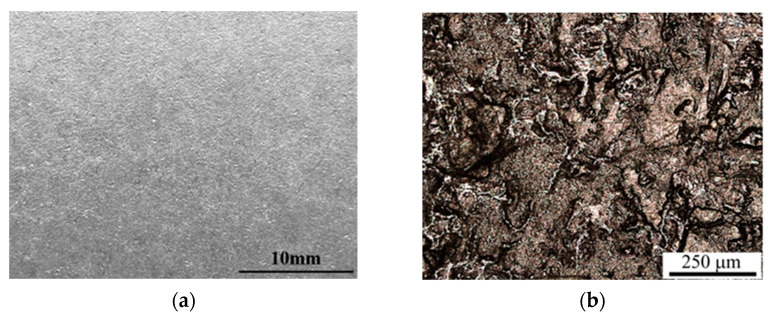
Macroscopic (**a**) and microscopic (**b**) observation of coating.

The results of macroscopic observations indicate that the Al-10%Si coating had a smooth and shiny surface with no signs of peeling and pock-marking (Figure 8a), meeting the requirements of the standard [62]. Evaluation of the coating surface was also carried out by measuring the basic surface roughness parameters. The average values of the surface roughness parameters Ra and Rz measured in the Al-10%Si coating surface (Figure 8b) are shown in Table 6. Therefore, the measured values are in the upper limit of the standardisation requirements (Table 1). Regardless of the side being measured, dispersion is small, which proves the high stability of the surface roughness of the Al-10% Si coating.

One of the most important parameters of the strip, determining corrosion resistance, and thus suitability for its use in exhaust system components, is the thickness of the Al-10%Si coating. The average coating thickness on side A is g_p_ = 18.8 µm, while, on side B, it is g_p_ = 22.7 µm (Table 6). The variation in the coating thickness is important in terms of manufacturing and use of the tubes. A large spread of measurements enforces continuous control of coating thickness after tube forming. A reduction in Al-10%Si coating thickness influences the risk of reducing corrosion resistance. One should pay attention that a thicker coating is on outer side of the tube during tube forming from strip since this is more exposed to the impact of weather conditions during operation.

**Figure 9:**
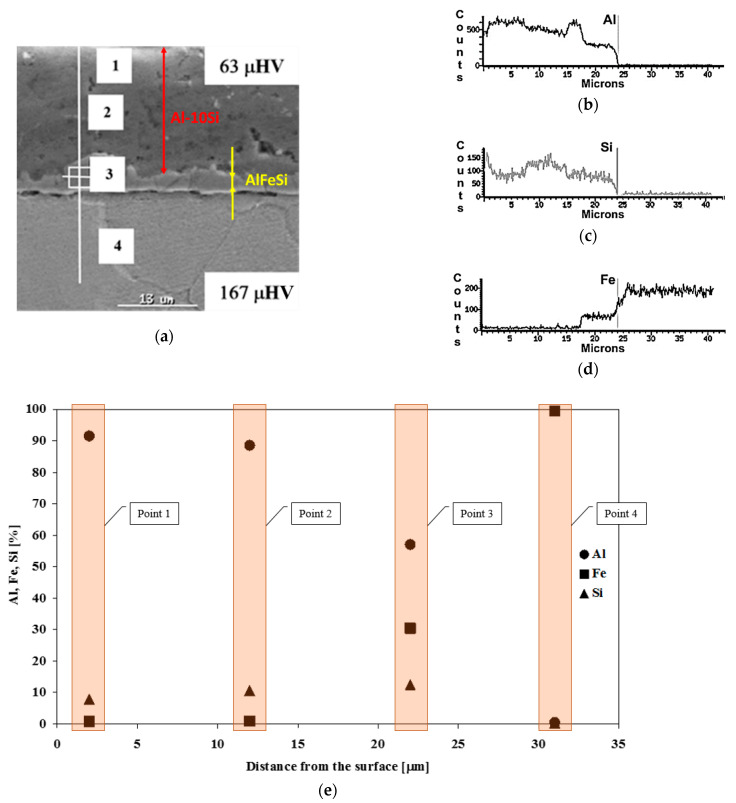
Results of linear (**a**–**d**) and point (**e**) chemical composition analysis of the coating.

Observations of the coating cross-section, results of microhardness, and the results of point and linear chemical composition microanalysis of the coating and the substrate are shown in Figure 9. In the initial state, the coating was a bilayer (Figure 9a). It consisted of a two-component coating Al-10%Si with a dominant aluminum content and three-component IL Fe-Al-10%Si, with a thickness representing approx. 20% of the Al-10%Si coating thickness, connecting the coating to the substrate. The division zone between the substrate and the IL was smooth. Al and Si content in the coating was 85–95% and 8–12%, respectively.

Local precipitation of silicon increased the share of this element in the zone analysed and reduced the Al content. The cause of these precipitations may be due to improper modification of the chemical composition of the Al-10%Si coating, and a lack of Si dispersion. In the IL, aluminum content was decreased below 60%, until its almost complete disappearance in the substrate material. Iron was not present in the coating, while its content in the IL was about 30% and about 100% in the substrate material (Figure 9b). Linear analysis of the chemical composition (Figure 9c) has shown a characteristic step change in the content of elements in the IL zone. The microhardness of the coating was on average 63 HV0.025, while, for the substrate, it was 167 HV2. The results indicated that the coating is very soft, which can lead to damage as the tube forms a strip.

### 3.2. Manufacturing Processes

#### 3.2.1. Cutting Process

**Figure 10:**
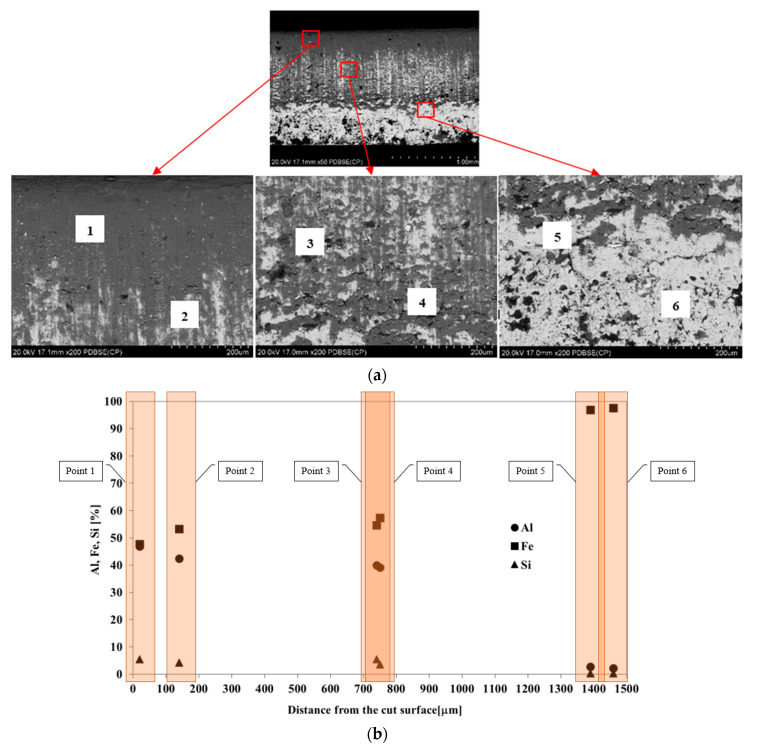
(**a**) Locations of the points for chemical composition analysis in the strip cross section and (**b**) results for points located in the top area (points 1, 2), centre area (points 3, 4) and bottom area (points 5, 6).

**Figure 11:**
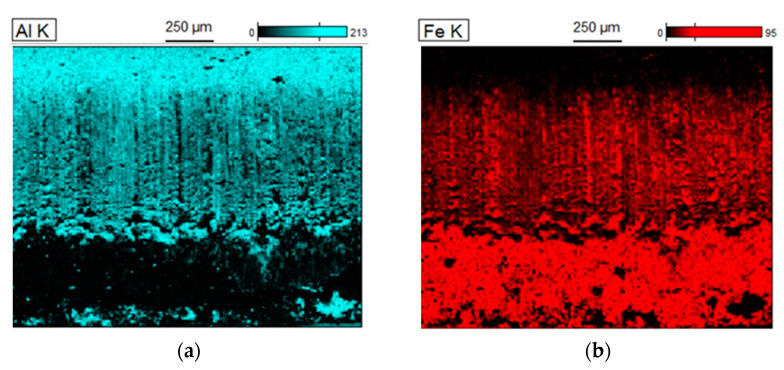
EDS mapping of the chemical composition of the strip cross-section surface; Al distribution (**a**), Fe distribution (**b**).

A cross-section of the strip after cutting was subjected to microscopic observations (Figure 10a). In the cutting process, the presence of Al-10%Si coating, which may occur as a result of pulling on the cut surface and affects the mechanical and plastic properties of the weld, plays a key role. In order to reveal whether there were no traces of pulling of Al-10%Si coating on the cut surface, microscopic observations and analysis of the chemical composition were made in the top, centre and bottom section of the cut strip (Figure 10b). Moreover, EDS mapping was carried out over the entire surface (Figure 11).

Al content is in the range 42–47% in the upper part of the sectional area of the strip (bend zone and plastic flow zone), 39–40% in the centre part (plastic flow zone) and 2–3% in the lower part of the strip (cracking zone). The content of Si was 3.5–5.5% in the upper and centre parts of the strip. In contrast, Fe content was 47–53% in the upper part of the strip cross-section, 55–57% in the centre and more than 95% in the lower part of the strip. The results of the point chemical composition (Figure 10b) and EDS mapping (Figure 11) indicate the presence of Al-10%Si coating on the surface of the cut strip. The coating is pulled to the bend zone and plastic flow zone, which may be one of the causes of defects in the tube joint after welding.

The average thickness of the coating on side A was g_p_ = 18.8 µm, while, on the B side, it was g_p_ = 22.7 µm. Values of average roughness Ra were in the range 1.8 (side A)–2.2 µm (side B) while the maximum height of the profile R_z_ was assessed to be 12 µm on side A and 14 µm on side B. The results show no effect of the cutting process on the surface roughness of the coating and coating thickness.

#### 3.2.2. Tube Welding Process

**Figure 12:**
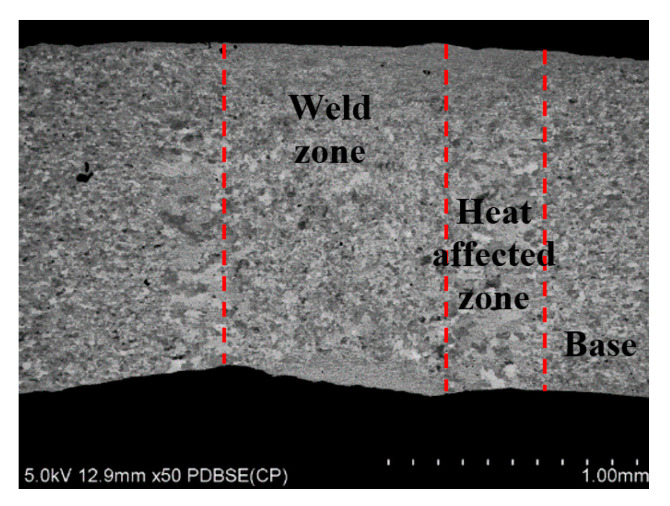
Microstructure of the seam zone, heat affected zone and the base material in the tube cross-section.

**Figure 13:**
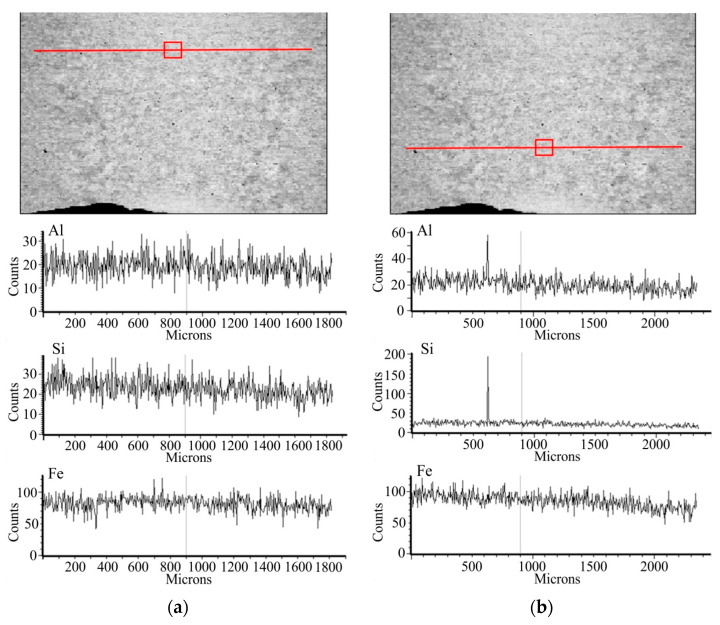
Chemical composition in the top (**a**) and bottom (**b**) of the weld area (linear analysis).

**Figure 14:**
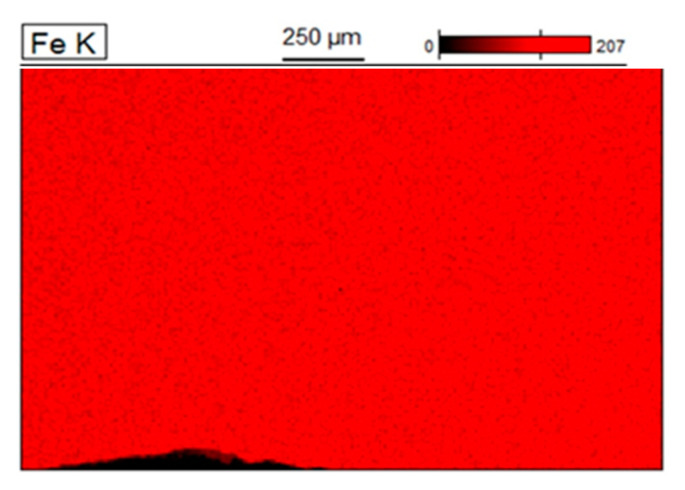
EDS mapping of the chemical composition of the weld area.

A quality assessment of the weld is performed after the welding process, before the removal of the flash. An example of an observation of the weld is shown in Figure 12. The grain size of the base material was 20–40 µm. However, in the weld zone and the heat affected zone (HAZ), this increases to 50–100 µm. The maximum value of microhardness of 285 HV2 occurs near the weld axis and then decreases to 163 HV2 at a distance of about 1.5 mm from the weld axis; this value is close to the microhardness of the charge material. Next, the study was focused on the exposure of the possible presence of a coating in the weld, resulting from improper implementation of the process of removing the coating from the edge of the strip. For this purpose, linear chemical composition analysis was carried out in several areas of the joint (Figure 13) as was EDS mapping over the whole area (Figure 14).

EDS mapping indicated the occasional occurrence of Al and Si in the chemical composition of the weld due to lack of precision in removing the coating. The presence of contaminants in the weld may cause the formation of defects in this area during the expansion or bending of the tubes. The average thickness of the coating on the outer surface of the tube, in the area outside the weld, is approx. 20 µm while the values of surface roughness parameters Ra = 1.2 μm and Rz = 5 μm indicate that the fusion process has no effect on the topography of the coating.

#### 3.2.3. Flash Removal Process

**Figure 15:**
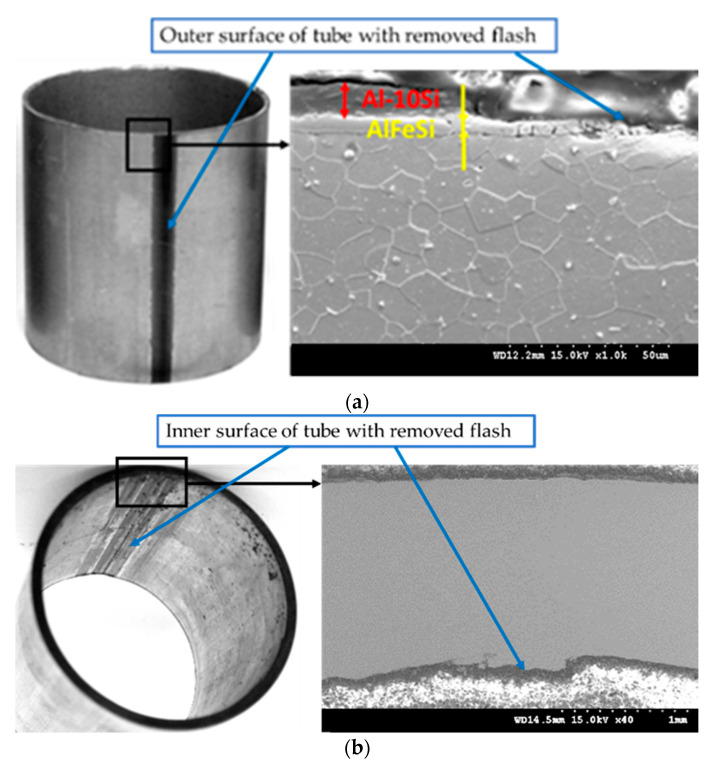
Outer (**a**) and inner (**b**) surface of tube with the flash removed.

Microscopic and macroscopic observations of tubes with the outer flash removed are shown in Figure 15a, while the tube with the inner flash removed is shown in Figure 15b. Improperly carrying out the process of removing the outer and/or inner flash may result in an additional Al-10%Si coating being removed from the outer and inner surface of the pipe in zones near the weld. The outer surface may be protected by an additional filling process of the coating on the area of the weld. However, the technical impossibility of an additional filling process of the weld area on the inner surface of the tube will result in the risk of corrosion.

#### 3.2.4. Process of Filling the Coating

**Figure 16:**
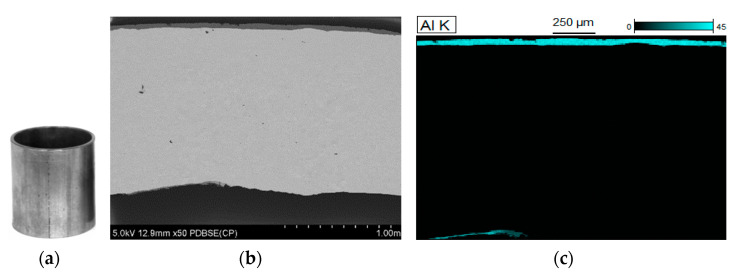
Observations of the sprayed coating on the weld: (**a**) macrograph, (**b**) SEM micrograph, (**c**) EDS mapping.

**Figure 17:**
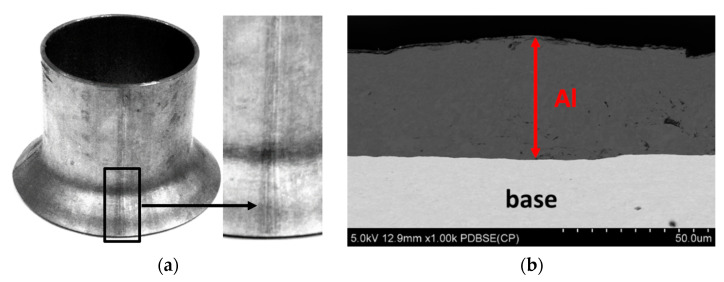
The observations of the sprayed coating on the weld after tube expansion: (**a**) macrograph, (**b**) SEM micrograph.

An evaluation of the quality of the sprayed coating was made based on macroscopic observation (Figure 16a), SEM micrographs (Figure 16b) and EDS mapping (Figure 16c). The thickness of the sprayed coating was in the range 25–30 µm. SEM micrographs show that the coating adheres tightly to the weld, while its structure is compact with no signs of cracking or porosity. Confirmation of good adhesion of the coating is supplemented by observations of the samples after expansion on the cone (Figure 17).

### 3.3. Investigations of Finished Tubes

The results of the tests on coating thickness after the calibration process are in the range g_p_ = 19.8–20 µm. The surface roughness parameters Ra = 1 µm, Rz = 4–5 µm indicate a minimal impact of the calibration process in changing these parameters. The test results of the calibration process with regard to shape, dimensions and properties of the finished tubes are shown in this section.

The next stage of the study was to evaluate the quality of welded aluminised steel tubes with an outer diameter D_z_ = 50 mm and wall thickness g_r_ = 1.5 mm. The measurements of the outer diameter of the tubes are in the range D_z_ = 49.92–50.09 mm. The average value and the median are D_z_ = 50.01 mm. The permissible dimensional tolerance is ±0.25 mm, so the tubes meet the requirements for outer diameter. The results of measurements of tube wall thickness are in the range g_r_ = 1.496–1.504 mm. The average value and the median are approx. g_r_ = 1.5 mm. Given that the tolerance is ±0.05 mm, the tubes meet the requirements for wall thickness. The wall thickness is also within the allowable dimensional tolerance of ±0.05 mm.

The results of the tests on the mechanical properties of the pipes showed differences in properties in the cross-section of the pipes. In the weld area, the material has the highest strength and the lowest elongation. At the point opposite the weld (180° from the weld), the elongation and UTS properties are comparable with the properties of the charge material; however, yield strength is significantly different. The yield strengths for a specimen cut from the tube and a strip specimen were YS_0.2_ = 228 MPa and YS_0.2_ = 170 MPa, respectively.

A slightly higher UTS-value was found in the samples cut at an angle of 30° (Figure 3) from the weld and at an angle of 90° from the weld. This suggests that the bending deformation is variable over the circumference and in such a way that, if the weld is at the top, the upper part of the tube circumference is the most deformed. Likewise, the lower half of the tube circumference is deformed to a smaller extent. The increase in strength properties is accompanied by a decrease in plastic properties. The results of the strength properties YS_0.2_ = 246 MPa, UTS = 305 MPa and elongations A_80_ = 30.5%, E_u_ = 21% show that the tube meets the production requirements.

**Figure 18:**
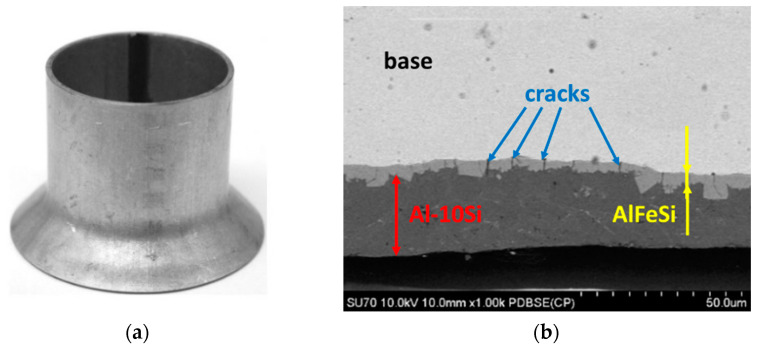
Observations of the coating after tube expansion: (**a**) macrograph, (**b**) SEM micrograph.

The height of the internal flash is in the range h_w_ = 0.2–0.3 mm, also meeting the requirements in this range. The surface of the tube is smooth and shiny with no signs of flaking and tears. Confirmation of good adhesion of the coating to the substrate is supplemented by observations of the samples after expansion on the cone (Figure 18). The results of the thickness tests of the coating on the outer surface of the tube indicate local thinning of the coating layer from 22.5 µm for the strip to approx. 19.8–20 µm on the finished product. Taking into account the production requirements, the coating thickness is satisfactory despite its reduction by about 10–15%. This suggests that using a batch of strip with a coating thickness of approx. g_p_ = 18 µm, the final local thickness will be around g_p_ = 15–16 µm, which is too low to meet the corrosion resistance requirements of the tube.

The surface roughness measurements show a decrease from Ra = 2 µm for the strip to approx. 1 µm on the finished product. The decrease in surface roughness provided smoothing of the coating surface, which is most desirable because of the minimisation of the microporosity of the coating, thereby lowering the stress concentration at the surface defects and increasing the strength of the material. The microhardness of the coating outside of the weld (65 HV0.025) and in the substrate (169 HV0.025) is comparable to that obtained in investigations of strips.

During tube expansion, the diameter of the tube in the greatest cross-section increased to a value of approx. D_u_ = 70 mm. This means a 50% increase in the initial diameter of the pipe. Non-uniform material flow during expansion of welded tubes was confirmed by varying the characteristics of the tube around the circumference. The coating of the tube showed a high plasticity and good adhesion. In addition, cracks in the substrate material were also observed at the edge of the greatest diameter.

### 3.4. Pipe Testing under Working Conditions

**Figure 19:**
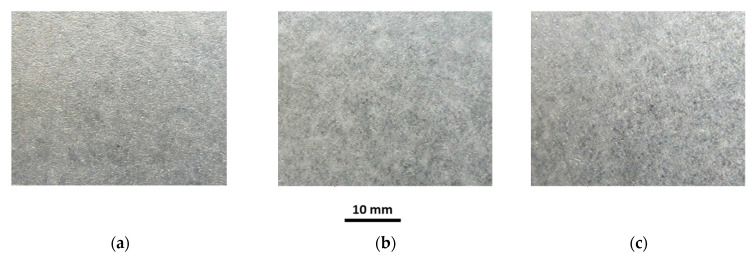
Macrographs of the coating surface after heat treatment at a temperature of 250 °C, for (**a**) 30 min, (**b**) 180 min, (**c**) 1440 min.

**Figure 20:**
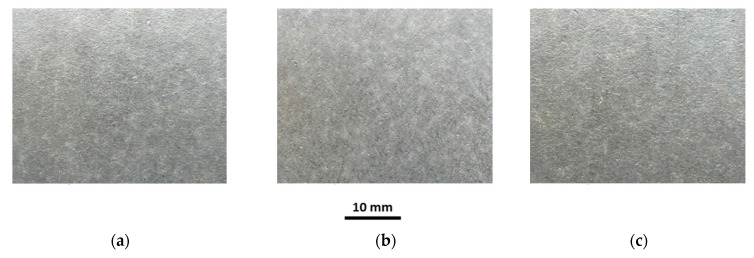
Macrographs of the coating surface after heat treatment at a temperature of 450 °C, for (**a**) 30 min, (**b**) 180 min, (**c**) 1440 min.

**Figure 21:**
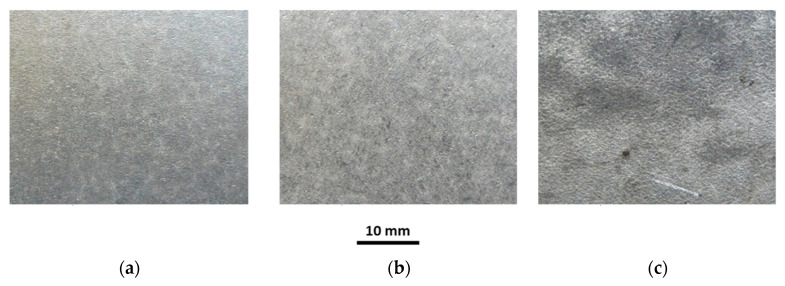
Macrographs of the coating surface after heat treatment at a temperature of 500 °C, for (**a**) 30 min, (**b**) 180 min, (**c**) 1440 min.

**Figure 22:**
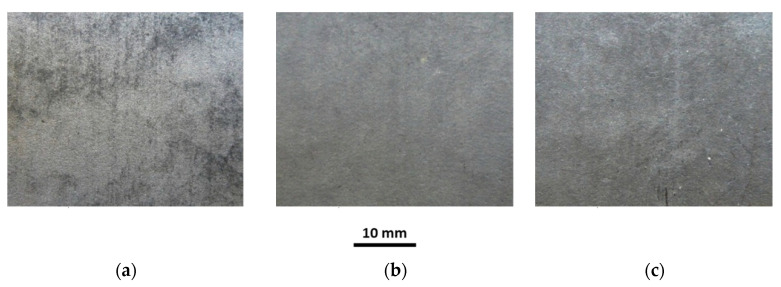
Macrographs of the coating surface after heat treatment at a temperature of 600 °C, for (**a**) 30 min, (**b**) 180 min, (**c**) 1440 min.

**Figure 23:**
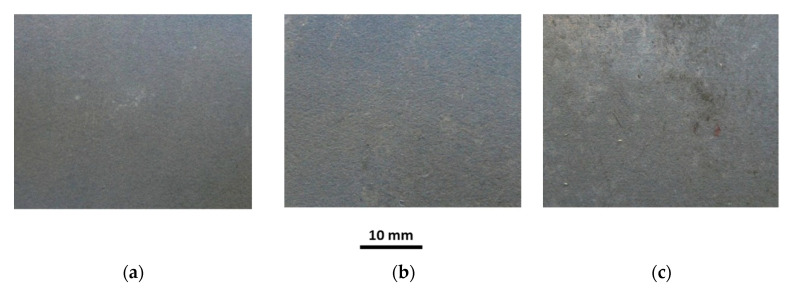
Macrographs of the coating surface after heat treatment at a temperature of 700 °C, for (**a**) 30 min, (**b**) 180 min, (**c**) 1440 min.

The investigations of tubes under working conditions covered the influence of temperature and sample annealing time on the surface roughness, coating thickness, chemical composition and microstructure of the coating. Selected macroscopic observations of the Al-10%Si coating surface after heat treatment at 250 °C and 700 °C for annealing times of 30, 180 and 1440 min are shown in Figure 19, Figure 20, Figure 21, Figure 22 and Figure 23.

The appearance of the coating surface depends on the temperature and heating time. The surface of the coating after heat treatment at a temperature of 450 °C for the full-time interval (Figure 19c), at a temperature of 500 °C for 30 min (Figure 21a) and 180 min (Figure 21b) and at a temperature of 550 °C for 30 min does not differ from the initial state of the coating. It is light and silvery with a distinct shine. The observations do not show any changes in the surface of the coating under the influence of temperature and annealing time. The surface of the coating has distinct, irregular, emphasised grains. On the other hand, after exposure to a temperature of 500 °C for 1440 min (Figure 21c), 550 °C for 180 min and 600 °C for 30 min (Figure 22a), in many areas, the coating changes to a darker and matt finish. Outside of these areas, the coating is bright but without shine. Observations indicate the disappearance of distinct grains on the coating surface in areas where the coating has become dark and matt. The tests have shown that this variation was due to the non-uniform thickness of the coating.

Reduced thickness of the coating produces darker and matt areas, while greater thickness of the coating ensures a brighter appearance of the coating. However, after heat treatment at a temperature of 550 °C for 1440 min, 600 °C for 180 min (Figure 22b) and in the range of 650–700 °C for an annealing time of 1440 min, the whole surface of the coating is dark, matt and porous, regardless of the thickness of the coating. Observations indicated the disappearance of distinct grains on the surface of the coating. The appearance of small brown-coloured areas on the coating surface in areas where the coating has the smallest thickness after annealing at 700 °C for 1440 min is to be noted. This testifies to the diffusion of iron from the substrate through the coating to the free surface.

**Figure 24:**
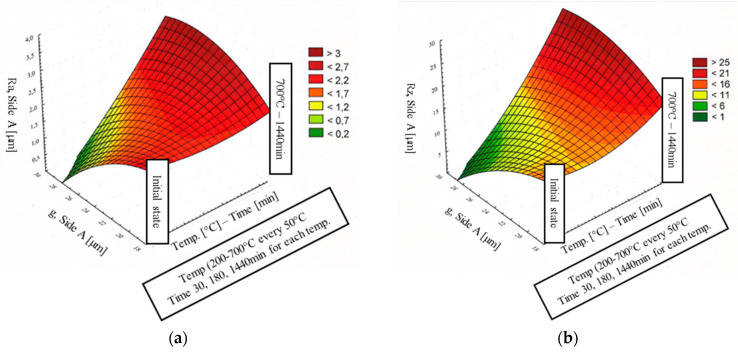
Effect of coating thickness and heat treatment parameters on the surface roughness parameters of coatings on the A side: Ra (**a**) and Rz (**b**).

**Figure 25:**
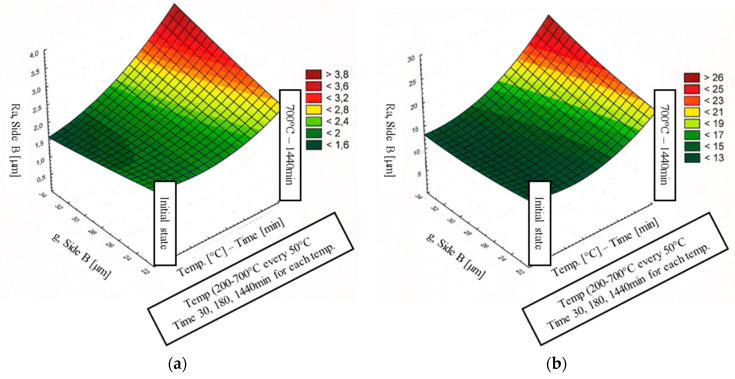
Effect of coating thickness and heat treatment parameters on the surface roughness parameters of coatings on the B side: Ra (**a**) and Rz (**b**).

The results of the correlation dependencies between the roughness and the thickness of the coating and the parameters of temperature and annealing time are presented in Figure 24a,b for the A side and Figure 25a,b for the B side.

At temperatures of 250–400 °C, the average thickness of the coating changes insignificantly over the entire annealing time range (max. g_p_ = 19.6 µm on the A side and g_p_ = 23.7 µm on the B side) compared to the starting material. At temperatures of 450–500 °C, the increase in the average thickness of the coating depends on the exposure time. For short annealing times (30 min and 180 min for 450 °C, and 30 min for 500 °C), the increase in the thickness of the coating is small, while longer annealing times (1440 min for 450 °C and 500 °C) increase the thickness of the coating (max. g_p_ = 28 μm). At a temperature of 550–700 °C over the entire annealing time range, the average coating thickness increases in relation to the initial coating thickness to a maximum of g_p_ = 26.7 µm on the A side and to g_p_ = 31.1 µm on the B side. The increase in the coating thickness depends on the time and temperature of annealing, and thus on the change in the appearance of the coating surface. The significant increase in the thickness of the coating occurs for an annealing temperature in the range 600–700 °C. At the same time, in this temperature-time interval, the coating surface becomes dark, matt and porous. The percent change in coating thickness on the A and B sides is approximately the same.

In the initial state, the surface roughness parameters of the coating Ra and Rz were 1.8 μm and 12 μm, respectively. In the temperature range of 250–600 °C, the Ra parameter ranges between 1.8 and 2.2 μm in almost the entire annealing time range, while the Rz parameter ranges between 12 and 14 μm. The exceptions are samples with darker areas or dullness. Then, Ra was approximately 2.4 μm and Rz was approximately 16 μm. In the temperature range of 650–700 °C, the mean roughness and maximum height of the assessed profile Rz increased to 2.5–3.3 μm and 19–23 μm, respectively. This is due to the change in the appearance of the surface of the coating from smooth, light and shiny to dark, rough, porous and matt.

**Figure 26:**
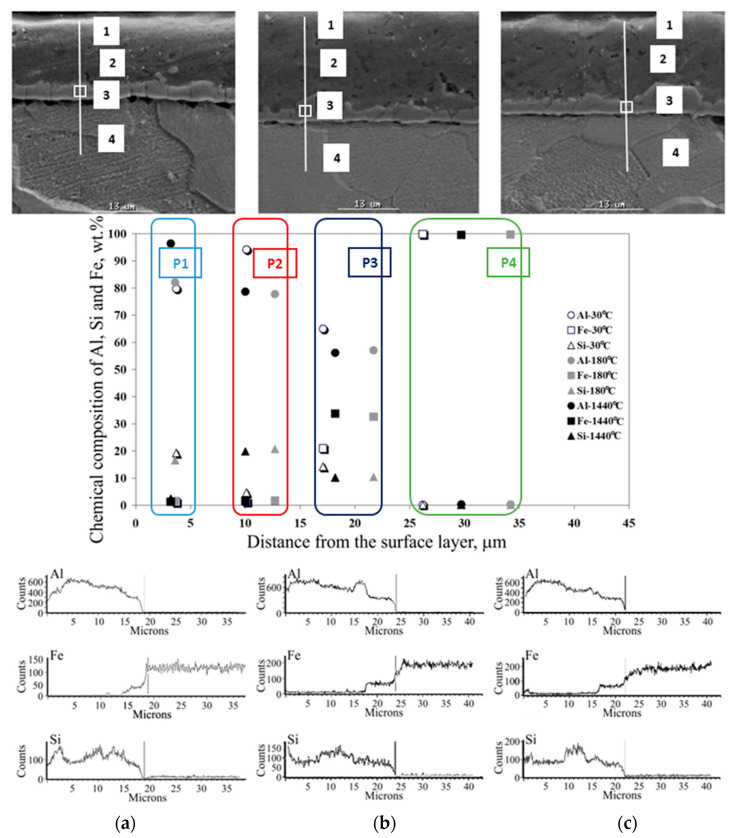
SEM micrographs and the results of point and linear EDS analysis of the chemical composition of the coating after heat treatment at a temperature of 250 °C during (**a**) 30 min, (**b**) 180 min, (**c**) 1440 min.

**Figure 27:**
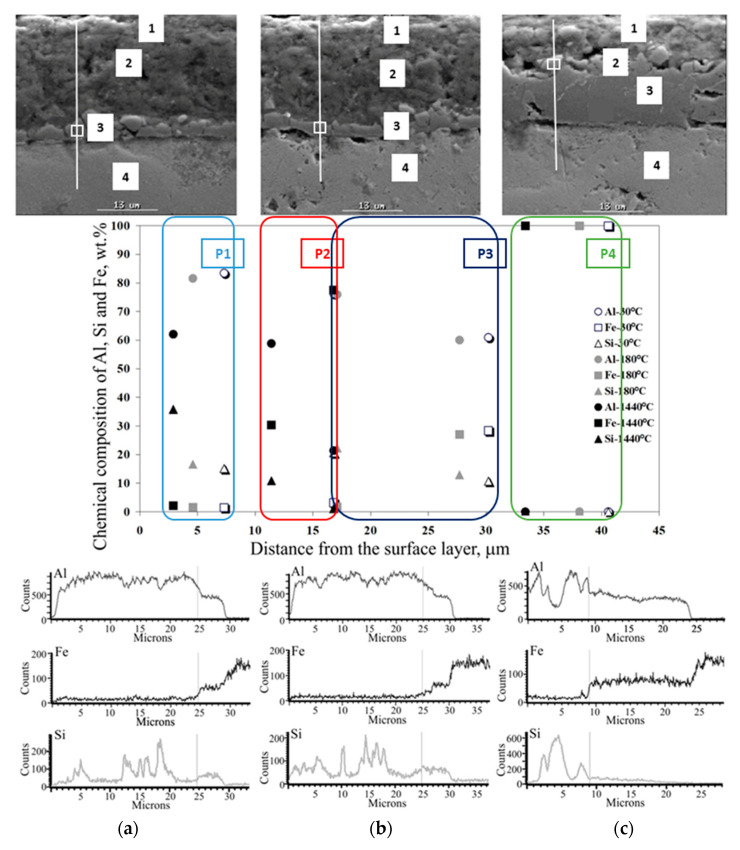
SEM micrographs and the results of point and linear EDS analysis of the chemical composition of the coating after heat treatment at a temperature of 450 °C during (**a**) 30 min, (**b**) 180 min, (**c**) 1440 min.

**Figure 28:**
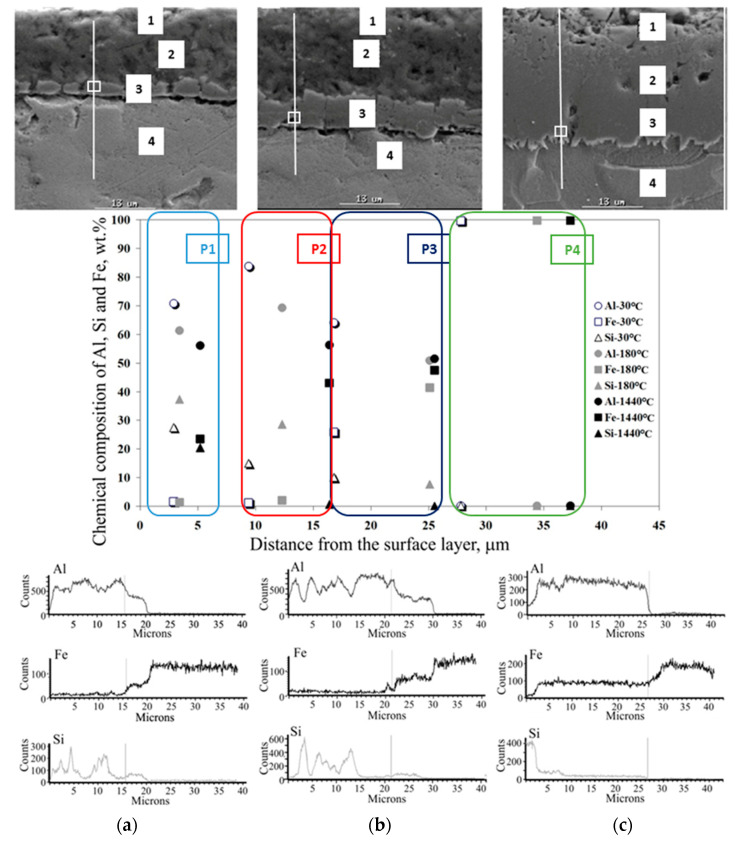
SEM micrographs and the results of point and linear EDS analysis of the chemical composition of the coating after heat treatment at a temperature of 500 °C during (**a**) 30 min, (**b**) 180 min, (**c**) 1440 min.

**Figure 29:**
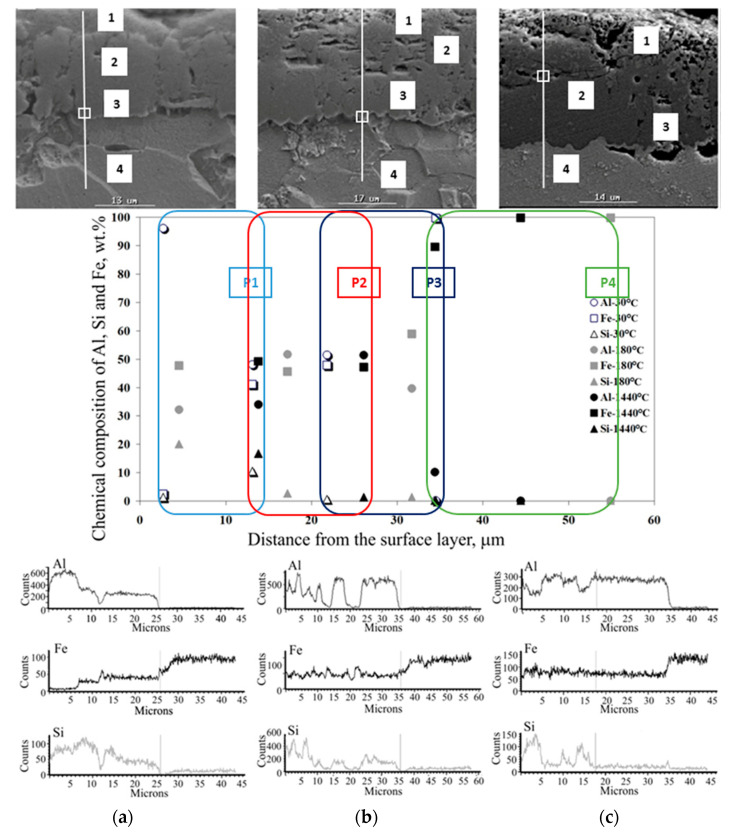
SEM micrographs and the results of point and linear EDS analysis of the chemical composition of the coating after heat treatment at a temperature of 600 °C during (**a**) 30 min, (**b**) 180 min, (**c**) 1440 min.

**Figure 30:**
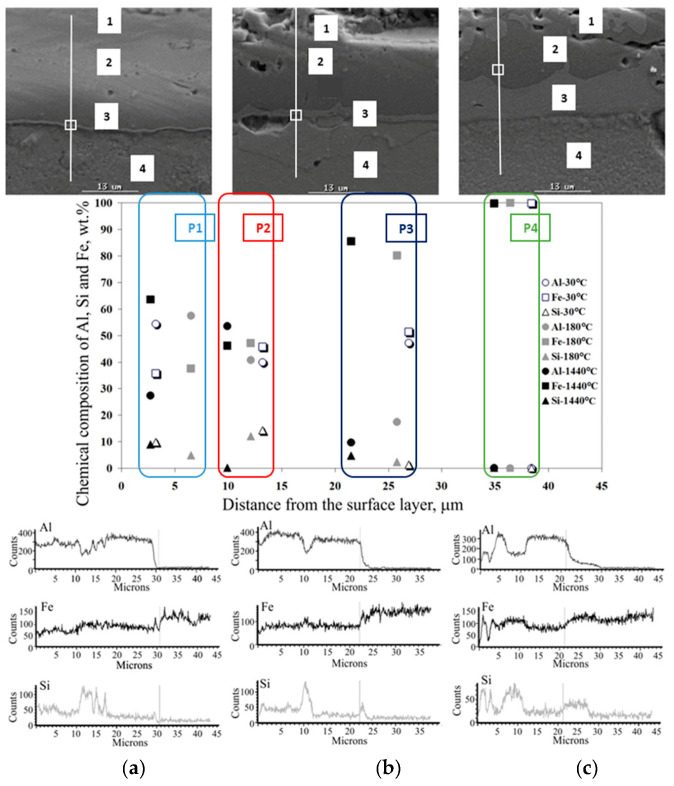
SEM micrographs and the results of point and linear EDS analysis of the chemical composition of the coating after heat treatment at a temperature of 700 °C during (**a**) 30 min, (**b**) 180 min, (**c**) 1440 min.

The effect of heat treatment is particularly visible in changes in the microstructure of the coating. The observations of the coating microstructure and the results of the point and linear analysis of the chemical composition on the samples after thermal treatment are shown in Figure 26, Figure 27, Figure 28, Figure 29 and Figure 30.

After heat treatment at a temperature of 250–350 °C (Figure 26) over the entire time range and at a temperature of 400 °C for 30 and 180 min, the Al-10%Si coating is a two-layer one. It consists of the characteristic Al-Fe-Si IL and the proper Al-Si coating. The IL, created as a result of the aluminising process that determines the good adhesion and corrosion resistance of the coating, is situated between the substrate and the coating. The Al content in the coating is 85–95% and is closely correlated with the Si content of 8–12%. Local precipitation of Si, increasing the share of this element in the zone analysed, reduces the Al content. There is no iron in the coating. On the other hand, in the IL, there is a clear increase in Fe content to approx. 30–40% and a simultaneous decrease in the Al content from 90% to approx. 50–60%, with the Si content unchanged. The linear EDS mapping shows a characteristic step-change in the content of elements in this zone. The iron content in the substrate material is approx. 100%. The coating, apart from the aforementioned local Si precipitates, is uniform and compact, with minimal areas of porosity. The IL, apart from small vertical cracks, adheres closely to the substrate. A smooth interface between the IL and the substrate is characteristic, whereas the phase boundary between the IL and the coating is smooth in some areas and in others has a stepped character. The free surface of the coating is smooth.

After annealing at 400 °C for 1440 min, 450 °C for 30 and 180 min (Figure 27a,b) and 500 °C for 30 min (Figure 29a), there is a change in the microstructure of the Al-Si coating. Characteristic several-micron Si precipitates occur over the entire area observed, confirmed by the results of linear EDS mapping of the chemical composition. On the other hand, the content of individual components in the coating and IL remains at a similar level to that after annealing at a lower temperature. The thickness of the IL also does not change. There are more vertical cracks in the IL, while the phase boundaries between the substrate, the IL and the coating are non-uniform, jagged and stepped. In the temperature range and annealing time examined, the IL adheres closely to the substrate. The free surface of the coating shows micro-irregularities.

After annealing at a temperature of 450 °C for 1440 min (Figure 27c) and at 500 °C for 180 min (Figure 28b), there are large Si precipitates in the microstructure of the coating across the entire area examined, which is confirmed by the results of linear EDS mapping. At the annealing temperature of 450 °C, the Al and Si content in the coating is approx. 60% and 10–35%, respectively. In the IL, the content of Al drops abruptly to the value of about 20%, Si to about 1%, while the content of Fe increases to almost 80%. At an annealing temperature of 500 °C, the Al and Si content in the coating is approx. 60–70% and 30–35%, respectively. In the IL, the Al content drops abruptly to about 50%, Si to about 10%, while the Fe content increases to about 40%. Obviously, Fe dominates in the substrate. Despite the lower temperature of the heat treatment—450 °C, a much longer heating time (1440 min) influences the intensification of Fe diffusion into the coating that is illustrated by the results of the EDS mapping and observations of the microstructure. In both cases, the increase in the thickness of the IL is characteristic, the microstructure of which has a few vertical cracks and the presence of small areas of microporosity. The phase boundaries between the substrate, the IL and the coating are non-uniform, jagged and stepped.

After annealing at a temperature of 550 °C for 30 min, there are small Si precipitates. The microstructure of the coating is compact without any porosity. The Al and Si content is 85–97% and up to 10%, respectively. In the intermetallic layer, there is a sharp decrease in the content of Al to the value of about 50%, a slight decrease in the Si content to the value of about 8% while the Fe content increases to a value of more than 40%. The microstructure that shows few vertical cracks is characteristic for the intermetallic layer. The phase boundaries between the substrate, the intermetallic layer and the coating show micro-irregularities. The high annealing temperature and a short exposure time determine that the Fe diffusion into the coating takes place earlier than the growth of Si precipitates. The free surface of the coating is slightly wavy.

After heat treatment at a temperature of 500 °C for 1440 min (Figure 28c), 550 °C during 180 and 1440 min, and 600 °C for 30 min. (Figure 29a), the Fe diffusion into the coating is very clear. The Al-10%Si coating constitutes approx. 20% of the total thickness of the coating and consists of about 96% of Al and the minimum content of Si and Fe. In the intermetallic layer, the content of Al drops abruptly to the value of about 50% and the Fe content increases to the value of about 50%, with a trace amount of Si. The free surface of the coating is characterized by waviness. The intermetallic layer constitutes the substantial contribution in the overall thickness of the coating. There are small areas of microporosity in the microstructure. In this case, the high annealing temperature and a short exposure time determine the Fe diffusion into the coating takes place earlier than the growth of Si precipitates. The interfaces between the substrate, the intermetallic layer and the coating are jagged.

After heat treatment at a temperature of 600 °C for 180 and 1440 min (Figure 29b,c) and at temperatures of 650 °C and 700 °C (Figure 30), the microstructure and chemical composition of the coating completely changes in the full range of time exposure. The two-phase intermetallic layer changes into a single-layer three-component coating. The content of Al was in the range of 30–50%, Fe in the range of 50–60%, and the content of Si was in the range of 1–20%. An increase in the Si content causes a decrease in Al content and vice versa. The abrupt change in the content of individual elements occurs only at the transition of the coating into the base material, which consists only of Fe. The free surface of the coating is non-uniform and jagged. There are numerous areas of porosity from the free surface to about half the thickness of the coating. The interface between the coating and the substrate is jagged. The coating tends to penetrate into the substrate material.

## 4. Conclusions

1. This article presents the results of a comprehensive study of the Al-10%Si coating on steel strip and tubes used for elements of exhaust systems. The comprehensive approach was expressed by versatile testing of the input materials in the form of sheets, analysis of the manufacturing process of welded pipes from these materials and testing of the finished products.
2. Research prevented the full identification of the initial state of the coating and strip. Due to the requirements resulting from the processing methods, the sheets and tubes used in exhaust systems must be subject to regular checks, in particular the stability of the thickness and width of the strip, the stability of the coating thickness, the adhesion of coating to the substrate and the mechanical properties.
3. Investigations on the effect of the conditions of the Al-10%Si aluminizing process and the process for tube manufacturing on the state and properties of the coating provided extensive material suitable for the design of these processes.
4. The analysis of the coating of tubes produced by welding has demonstrated that, under process conditions, there is no degradation or cracking, the coating has full adhesion and suitable calibration tools and the working conditions provided the required surface quality of the tubes.
5. The results show that it is possible to obtain high quality welded aluminized steel tubes in a complex and controlled manufacturing processes, on the condition of taking into account the effect of the Al-10% Si coating on the conditions of these processes and the influence of these processes on the coating.
6. Tests simulating operating conditions have shown a significant effect of temperature and annealing time on the surface roughness, chemical composition and microstructure of the Al-10%Si coating on tubes.

## Figures and Tables

**Table 1:** Requirements regarding the chemical composition (wt.%) of DX53D + AS120 steel flat products.

C	Mn	P	S	Al	Fe
max 0.08	max 0.4	max 0.03	max 0.03	max 0.04	rest

**Table 2:** Requirements for basic mechanical properties of DX53D + AS120 steel flat products.

Yield Stress YS_0.2_, MPa	Ultimate Tensile Stress (UTS), MPa	Total Elongation A_80_, %	Thickness g_t_, mm	Al-10%Si Coating Thickness g_p_, µm	Average Roughness of Al-10%Si Coating Ra, µm
140–300	270–420	min. 28	1.5 ± 0.05	15–25	0.8–2.2

**Table 3:** Chemical composition of EN AW-1070A metallizing wire (wt.%).

Fe	Si	Cu	Zn	Ti	V	Cr	Mn	Mg	Al
0.12	0.07	0.002	0.008	0.001	0.001	0.001	0.002	0.002	rest

**Table 4:** The requirements for welded tubes DX53D + AS120.

Yield Stress R_p0.2_, MPa	Ultimate Tensile Stress R_m_, MPa	A_80_, %	Outer Diameter D_z_, mm	Wall Thickness g_r_, mm	Height of Inner Fin h_w_, mm	Thickness of Al-10%Si Coating g_p_, µm	Surface Roughness of Al-10%Si CoatingRa, µm
max 300	270–420	min 28	50 ±0.25	1.5 ±0.05	max. 0.3	18–25	0.8–2.2

**Table 5:** The chemical composition of the substrate of the aluminized steel strip (wt.%).

C	Mn	Si	P	S	Cr	Ni	Nb	Cu	Al	Fe
0.017	0.14	0.006	0.008	0.010	0.02	0.022	0.01	0.011	0.012	rest

**Table 6:** Surface roughness parameters and thickness of the Al-10%Si coating.

Side	Ra, μm	Rz, μm	g_p_, mm
A	1.8	12	18.8
B	2.2	14	22.7

## Data Availability

The data presented in this study are available on request from the corresponding author.
